# Plasma Markers of Neutrophil Extracellular Trap Are Linked to Survival but Not to Pulmonary Embolism in COVID-19-Related ARDS Patients

**DOI:** 10.3389/fimmu.2022.851497

**Published:** 2022-03-17

**Authors:** Renaud Prével, Annabelle Dupont, Sylvie Labrouche-Colomer, Geoffrey Garcia, Antoine Dewitte, Antoine Rauch, Julien Goutay, Morgan Caplan, Elsa Jozefowicz, Jean-Philippe Lanoix, Julien Poissy, Etienne Rivière, Arthur Orieux, Denis Malvy, Didier Gruson, Loic Garçon, Sophie Susen, Chloé James

**Affiliations:** ^1^ CHU Bordeaux, Medical Intensive Care Unit, Pessac, France; ^2^ Univ. Bordeaux, INSERM, U1045, Centre de Recherche Cardio-Thoracique de Bordeaux, Pessac, France; ^3^ Univ. Lille, INSERM, CHU Lille, Department of Hematology and Transfusion, Pôle de Biologie Pathologie Génétique, Institut Pasteur de Lille, UMR1011-EGID, Lille, France; ^4^ Univ. Bordeaux, INSERM, UMR1034, Biology of Cardiovascular Diseases, Pessac, France; ^5^ CHU Bordeaux, Laboratory of Hematology, Pessac, France; ^6^ Laboratoire d’Hématologie, CHU Amiens, EA4666 HEMATIM, UPJV, Amiens, France; ^7^ CHU Bordeaux, Department of Anaesthesia and Critical Care, Magellan Medico-Surgical Centre, Bordeaux, France; ^8^ Univ. Bordeaux, CNRS, UMR 5164, INSERM ERL1303, Immunology from Concept and Experiments to Translation (ImmunoConcEpT), Bordeaux, France; ^9^ Centre Hospitalier Universitaire Lille, Intensive Care Department, Pôle de Réanimation, Lille, France; ^10^ Centre Hospitalier Universitaire Lille, Surgical Critical Care, Department of Anesthesiology and Critical Care, Lille, France; ^11^ CHU Amiens-Picardie, Infectious Diseases Department, Amiens, France; ^12^ EA4294, Université Picardie Jules Verne, Amiens, France; ^13^ Univ. Lille, INSERM U1285, CHU Lille, Pôle de réanimation, CNRS, UMR 8576-UGSF-Unité de Glycobiologie Structurale et Fonctionnelle, Lille, France; ^14^ CHU Bordeaux, Internal Medicine and Infectious Diseases Unit, Pessac, France; ^15^ Department for Infectious and Tropical diseases, University Hospital Centre and INSERM 1219, University of Bordeaux, Bordeaux, France

**Keywords:** neutrophil extracellular trap, acute respiratory distress syndrome, COVID-19, immunothrombosis, pulmonary embolism

## Abstract

**Introduction:**

Coronavirus disease 2019 (COVID-19) can cause life-threatening acute respiratory distress syndrome (ARDS). Recent data suggest a role for neutrophil extracellular traps (NETs) in COVID-19-related lung damage partly due to microthrombus formation. Besides, pulmonary embolism (PE) is frequent in severe COVID-19 patients, suggesting that immunothrombosis could also be responsible for increased PE occurrence in these patients. Here, we evaluate whether plasma levels of NET markers measured shorty after admission of hospitalized COVID-19 patients are associated with clinical outcomes in terms of clinical worsening, survival, and PE occurrence.

**Patients and Methods:**

Ninety-six hospitalized COVID-19 patients were included, 50 with ARDS (severe disease) and 46 with moderate disease. We collected plasma early after admission and measured 3 NET markers: total DNA, myeloperoxidase (MPO)–DNA complexes, and citrullinated histone H3. Comparisons between survivors and non-survivors and patients developing PE and those not developing PE were assessed by Mann–Whitney test.

**Results:**

Analysis in the whole population of hospitalized COVID-19 patients revealed increased circulating biomarkers of NETs in patients who will die from COVID-19 and in patients who will subsequently develop PE. Restriction of our analysis in the most severe patients, i.e., the ones who enter the hospital for COVID-19-related ARDS, confirmed the link between NET biomarker levels and survival but not PE occurrence.

**Conclusion:**

Our results strongly reinforce the hypothesis that NETosis is an attractive therapeutic target to prevent COVID-19 progression but that it does not seem to be linked to PE occurrence in patients hospitalized with COVID-19.

## Introduction

Coronavirus disease 2019 (COVID-19) is responsible for more than 4,550,00 deaths worldwide at the beginning of September 2021 according to the World Health Organization (WHO). The vast majority of the infected people have subclinical to moderate forms, but some of them develop respiratory failure with acute respiratory distress syndrome (ARDS) (1). *Postmortem* histological analysis from COVID-19-related ARDS non-survivor patients exhibited vascular microthrombi in lung capillaries that participate in lung damage (2). Moreover, about 20% of critically ill COVID-19 patients develop pulmonary embolism (PE), which can aggravate their pulmonary condition and impair oxygenation because of shunt effect (3). Immunothrombosis is a physiological innate immune response that leads to formation of thrombi inside blood vessels in order to contain and destroy pathogens such as bacteria, fungi, and viruses (4). It involves neutrophils, monocytes, platelets, and activation of hemostasis. Activation of neutrophils by pathogens causes the emission of neutrophil extracellular traps (NETs) that are DNA fragments decorated with proteins of neutrophil origin such as myeloperoxidase (MPO) (5). When uncontrolled, immunothrombosis becomes detrimental to the host. As NETs are procoagulant (6, 7) and cytotoxic for lung vascular endothelial cells (8, 9), increased NETosis has been found to participate in various pathological processes such as arterial and venous thrombosis (6), ARDS (10), and other critical conditions not linked to COVID-19 (11).

It is now well-admitted that circulating markers of NET formation are associated with COVID-19 severity (12–14). Whether measurement of NET biomarkers early after admission for COVID-19 can be of prognostic value is a major question, as it would strengthen the rationale to target NETs and may help in clinical decision-making. The aim of this study is thus to evaluate whether plasma levels of NET markers measured shorty after admission of hospitalized COVID-19 patients are associated with clinical outcomes in terms of clinical worsening, survival, and PE occurrence.

## Patients and Methods

### Study Design and Participants

A prospective observational study was conducted in 3 French university hospitals from April to July 2020. We enrolled all patients with laboratory-confirmed COVID-19 admitted to conventional hospitalization ward (moderate, i.e., non-ARDS patients) and patients admitted to intensive care unit (ICU) for COVID-19-related ARDS (critical illness) defined according to National Institutes of Health treatment guidelines (15) with available samples. ARDS was defined according to Berlin’s criteria (16), and criteria for admission to ICU were persistence of SpO_2_ <92% and/or clinical respiratory failure despite conventional oxygen therapy. All ARDS patients required high-flow nasal cannula oxygen (flow between 30 and 60 L/min) or mechanical ventilation. Patients could only be included in the moderate or ARDS group for the subgroup analyses according to their clinical condition at admission to hospital. COVID-19 was defined as a positive result of real-time reverse transcriptase–polymerase chain reaction (RT-PCR) on nasal and pharyngeal swabs according to the WHO guidance. All patients received prophylactic heparin treatment according to the Groupe Français d'étude sur l'Hémostase et la Thrombose (GFHT) / Groupe d'Intérêt en Hémostase Périoperatoire (GIHP) proposals (17) or therapeutic treatment if indicated by their comorbidities, but patients with PE at the time of sampling were not included. PE was diagnosed by computerized tomography angiography performed at clinician’s discretion according to routine care. Routine criteria for receiving a computerized tomography angiogram in patients with ARDS were hypoxemia not improving with positive end-expiratory pressure titration or PaO_2_ worsening without lung compliance impairment or elevation of right heart pressure without lung compliance worsening. Ten non-hospitalized, non-COVID-19, healthy participants with no history of thromboembolic events, hemorrhagic events, or pneumonia were included as a control reference group.

### Data Collection

Data were prospectively recorded by physicians in charge of the patient by questioning the patients, patients’ family, and patients’ general practitioners. Electronic worksheet was completed by physicians caring for the patients.

### Sample Collection

Samples were collected early after admission to hospital for moderate COVID-19 patients and at admission to ICU for ARDS patients. Both non-ICU COVID-19 patients and COVID-19 ARDS patients were included at direct admission or after a short (<12 h) stay in the emergency room. It implies that hospitalized patients who were further admitted to ICU were not included again in the ARDS patient group. Plasma samples were prepared from citrated blood after two 10-min centrifugations at 2,500g and stored at -80°C.

### Quantification of Plasmatic Cell-Free DNA

The Quant-it™ PicoGreen assay kit (Invitrogen, San Diego, CA, USA) was used to quantify circulating cell-free double-strand DNA according to manufacturer’s instructions. Fluorescence intensity was measured using a microplate photometer (Infinite^®^ 200 PRO NanoQuant Multimode Microplate Reader, Tecan; 480 nm excitation wavelength/523 nm emission wavelength).

### Quantification of Myeloperoxidase–DNA Complexes

MPO–DNA complexes were quantified by enzyme-linked immunosorbent assay (ELISA) using a modified approach of the Cell Death Detection ELISA kit (Roche, Basel, Switzerland) and the capture of anti-MPO antibody (Bio-Rad^®^) (7). To limit the inter-assay variability and because no international standard preparation is available to measure MPO–DNA complexes, we used a calibration range made from a stock solution of NETs, and results are expressed as standard NETs (ST) (18). The detailed protocol is available in the **Supplementary Material**.

### Quantification of Citrullinated Histone H3

Citrullinated histone H3 (H3Cit) was quantified with a slight modification of the ELISA previously described by Thalin et al. (19) by using Cell Death detection kit without streptavidin-precoated wells. The optical densities (ODs) were measured at a wavelength of 450 nm with a reference correction wavelength at 620 nm using a microplate photometer (Infinite^®^ 200 PRO NanoQuant Multimode Microplate Reader, Tecan).

### Statistical Analyses

No statistical sample size calculation was performed *a priori*, and sample size was equal to the number of patients admitted for COVID-19 with available frozen plasma. Continuous variables are presented as median and interquartile range (IQR) and are compared using the Mann–Whitney test for comparison between two groups and Kruskal–Wallis test with Dunn’s multiple comparison test for comparison between three groups. Categorical variables are expressed as the number of patients (percentage) and are compared using Fisher’s exact test. Correlation analysis was performed using Pearson correlation test. All analyses were performed on Prism 6.0 software (GraphPad, La Jolla, CA) and R 3.6.1 statistical software (R Foundation for Statistical Computing, Vienna, Austria).

### Ethics Statement

According to French law and the French Data Protection Authority, the handling of these data for research purposes was declared to the Data Protection Officer of the University Hospital of Bordeaux. Patients or relatives were notified about the anonymized use of their healthcare data *via* the departments’ booklets, and non-opposition was recorded. All patients included in the study gave their written informed consent for the use of their plasma. The study complied with the Declaration of Helsinki of 1975, revised in 2000. This study was approved by the French institutional authority for personal data protection [Commission Nationale de l’Informatique et des Libertés (CNIL), registration number DEC20-086] and ethics committee (ID-CRB 2020-A00763-36) and by the institutional review board of the University Hospital of Bordeaux (declaration number CE-GP-2020-39). Samples from healthy controls were authorized by the Comité de Protection des Personnes Sud Ouest et Outre Mer III DC 2015/94.

## Results

### Patients’ Characteristics

Ninety-six COVID-19 patients were included, 50 (52%) with ARDS and 46 (48%) with moderate disease. Samples were collected with a median delay from admission of 2 days [1-3] for moderate COVID-19 patients and of 1 day [1-2] for critical COVID-19-related ARDS patients. Patients’ characteristics are summarized in **Table 1**. Briefly, patients were mostly men (54% for moderate patients and 68% for ARDS patients) with a median age of 68 years for moderate patients and 61 for ARDS patients. Hypertension and diabetes were the main comorbidities (**Table 1**). PE occurred in 19 patients, one with moderate disease and 18 with ARDS. Death occurred in 29/96 patients (4/46 moderate patients and 25/50 ARDS patients). All patients were on heparin treatment at the time of blood sampling, and majority were on low-molecular weight heparin (LMWH) (66%). Compared to moderate patients, ARDS patients were more frequently treated with unfractionated heparin (UFH) than with LMWH (64% vs. 13%, p = 0.0001) and on therapeutic rather than prophylactic anticoagulant regimen (36% vs. 15%, p = 0.005). Median anti-Xa value for patients receiving UFH at therapeutic dose (n = 13) was 0.34 (IQR 0.195–0.515), with no difference between moderate and ARDS patients [respectively 0.35 (IQR 0.19–0.52) and 0.34 (IQR 0.21–0.49), p = 0.87). Indications for therapeutic anticoagulation at the time of sampling were atrial fibrillation (n = 9), history of phlebitis (n = 3), history of PE (n = 2), essential thrombocythemia with history of PE (n = 1), early initiation of veno-venous extracorporeal membrane oxygenation (ECMO) (n = 1), physicians’ discretion in front of elevated fibrinogen and/or D-dimer levels (n = 6), and unknown (n = 3). Nine patients were treated with veno-venous ECMO during their stay in the ICU.

**Table 1:** Patients’ characteristics at the time of blood sampling.

	Moderate COVID-19 patients	COVID-19 ARDS patients		p-value
	N = 46	N = 50		
Age (years)	68 [62–78]	61 [54–68]		0.008
Sex (male)	25 (54%)	34 (68%)		0.18
Body mass index (kg/m²)	26 [24–30]	29 [26–36]		0.008
Hypertension	29 (63%)	29 (58%)		0.82
Diabetes mellitus	8 (17%)	16 (32%)		0.19
Chronic kidney disease	3 (7%)	0 (0%)		0.25
Chronic heart disease	8 (17%)	9 (18%)		1.00
Chronic obstructive pulmonary disease	5 (10%)	10 (20%)		0.35
Immunosuppressive drug before COVID-19	6 (14%)	10 (20%)		0.55
Respiratory rate (/min)	24 [18–22]	30 [24–35]		0.04
Oxygen flow (L/min)	4 [2–6]	–		–
FiO_2_ (%)	–	60 [45–90]		–
Heart rate (/min)	97 [78–123]	105 [68–137]		0.90
Mean blood pressure (mmHg)	72 [64–91]	68 [59–94]		0.82
Temperature (°C)	37 [36.8–37.3]	38 [37–39]		0.08
Fibrinogen (g/L)	6 [5.45–6.5]	7.5 [5.8–8.5]		0.22
D-dimers (mg/L)	625 [513–838]	2,123 [1,230–6,308]		<0.001
Platelets (/mm^3^)	175,000 [111,250–252,500]	210,000 [130,000–320,000]		0.78
Neutrophils (/mm^3^)	3,300 [2,510–5,450]	6,300 [4,430–11,750]		0.02
Lymphocytes (/mm^3^)	1,000 [700–1,315]	820 [400–1,240]		0.07
C-reactive protein (mg/L)	49 [27–78]	132 [73–256]		<0.01
Anticoagulant treatment at the time of blood sampling				
Prophylactic vs. therapeutic anticoagulation regimen	39 (85%)/7 (15%)	32 (64%)/18 (36%)		0.05
Care during hospitalization				
Corticosteroids use	5 (11%)	16 (32%)		0.01
Immunomodulating agents	2 (5%)	8 (16%)		0.13
Tocilizumab	0	6		–
Anakinra	1	0		–
Interferon-β	1	0		–
Antiviral agents	3 (7%)	18 (36%)		0.001
Lopinavir-ritonavir	2	10		–
Remdesivir	1	1		–
Oseltamivir	0	2		–
Clinical evolution after blood sampling				
PE	1	18	
In-hospital mortality	4	25	

Continuous variables are presented as median and interquartile range and are compared using Mann–Whitney test. Categorical variables are expressed as the number of patients (percentage) and are compared using Fisher’s exact test.

ARDS, acute respiratory distress syndrome; FiO2, fractional inspired oxygen; PE, pulmonary embolism.

### Plasma Levels of Neutrophil Extracellular Traps Increase With COVID-19 Clinical Severity

**Figure 1:**
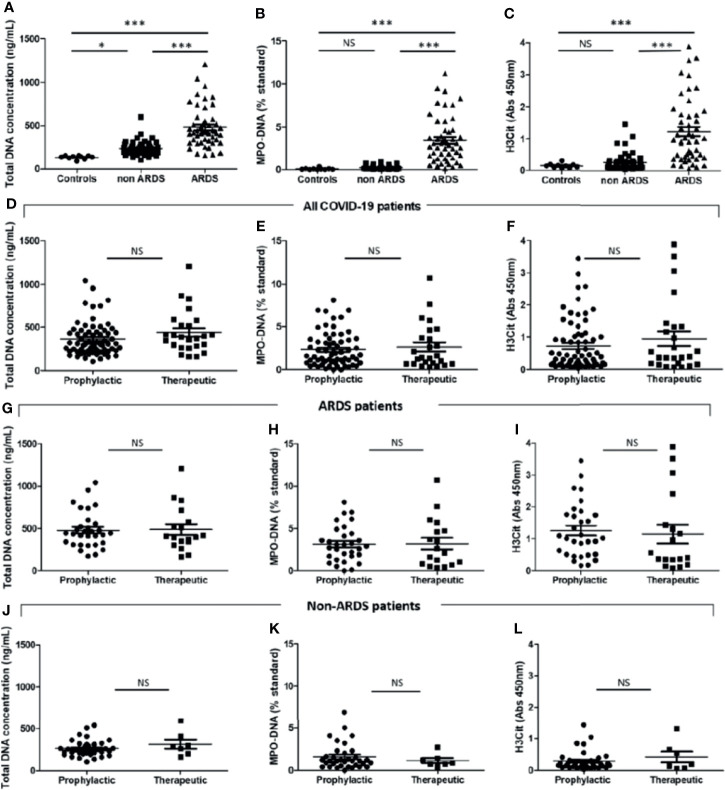
Critically ill COVID-19 patients have higher plasma neutrophil extracellular trap (NET) levels than those of moderate COVID-19 patients and controls. Plasma NET levels were compared between healthy donors (“controls”, n = 10), moderate [without acute respiratory distress syndrome (ARDS) “non-ARDS”] COVID-19 patients (n = 46), and patients with ARDS (n = 50) admitted to the intensive care unit. Levels of NET markers were as follows: plasma total DNA concentrations [140 ng/ml (124–151) vs. 220 ng/ml (188–271) vs. 428 ng/ml (324–560), p < 0.0001], plasma MPO–DNA levels [0.044 ST (0.012–0.093) vs. 10.15 ST (0.10–0.31) vs. 3.00 (1.38–4.63), p < 0.0001] and plasma H3Cit levels [0.14 (0.088–0.18) vs. 0.17 (0.10–0.30) vs. 0.97 (0.41–1.75), p < 0.0001]. **(A)** Total DNA concentration (ng/ml). **(B)** Myeloperoxidase–DNA levels (% standard NETs). **(C)** Histone H3 citrullinated (absorbance 450 nm). Plasma NET levels were compared between COVID-19 patients treated with prophylactic (n = 71) or therapeutic (n = 25) heparin treatment. **(D)** Total DNA concentration (ng/ml). **(E)** Myeloperoxidase–DNA levels (% standard NETs). **(F)** Histone H3 citrullinated (absorbance 450 nm). Plasma NET levels were compared between COVID-19-related ARDS patients treated with prophylactic (n = 32) or therapeutic (n = 18) heparin treatment. **(G)** Total DNA concentration (ng/ml). **(H)** Myeloperoxidase–DNA levels (% standard NETs). **(I)** Histone H3 citrullinated (absorbance 450 nm). Plasma NET levels were compared between COVID-19 moderate patients treated with prophylactic (n = 39) or therapeutic (n = 7) heparin treatment. **(I)** Total DNA concentration (ng/ml). **(J)** Myeloperoxidase–DNA levels (% standard NETs). **(K)** Histone H3 citrullinated (absorbance 450 nm). Threshold for statistical significance was a p-value of 0.05. *p < 0.05, ***p < 0.0001. NS, statistically non-significant.

We quantified 3 NET markers in patients’ plasma collected shortly after patients’ admission: one unspecific, i.e., total cell-free DNA, and two more specific, i.e., MPO–DNA complexes and H3Cit. For all 3 markers, we observed that all COVID-19 patients (n = 96) have significantly more NETs than healthy donors (n = 10), respectively: total cell-free DNA concentrations [304 ng/ml (209–443) vs. 140 ng/ml (124–151), p < 0.0001], plasma MPO–DNA levels [0.63ST (0.15–3.10) vs. 0.044 ST (0.012–0.093), p < 0.0001], and plasma H3Cit levels [0.37 (0.16–1.06) vs. 0.14 (0.088–0.18), p < 0.01]. The 3 NET markers were significantly higher in ARDS patients compared to patients with moderate COVID-19 disease (**Figures 1A–C**). Because one study reported that heparin can dismantle already formed NETs by removing histones from secreted DNA (20), we wondered whether heparin anticoagulation, either at prophylactic or therapeutic dose, modified NET dosages in COVID-19 patients. A first, analysis in all COVID-19 patients showed no difference in plasma levels of NET markers (total DNA, MPO—DNA, and H3Cit, respectively; **Figures 1D–F**) between patients treated with prophylactic or therapeutic anticoagulant treatment. As we were concerned that the more severe patients were the ones who received the most therapeutic anticoagulation, thereby inducing a bias in the analysis, we analyzed moderate and ARDS patients separately. We did not observe any difference of NET markers whether patients were under prophylactic or therapeutic heparin either for ARDS or moderate patients (respectively; **Figures 1G–L**).

### Levels of Neutrophil Extracellular Trap Markers at the Time of Admission Are Not Higher in Moderate COVID-19 Patients Who Will Later Worsen Their Respiratory Condition Compared With Those Who Will Not

**Figure 2:**
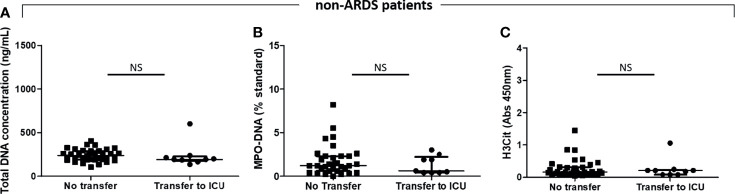
Plasma NET levels are not different between COVID-19 moderate patients who will subsequently need a transfer to the ICU than those who will not. Plasma NET levels were compared between moderate COVID-19 patients who will subsequently have a worsened respiratory condition needing a transfer to the intensive care unit because of acute respiratory distress syndrome (ARDS) (n = 9) and those who will not (n = 37). **(A)** Total DNA concentration (ng/ml). **(B)** Myeloperoxidase–DNA levels (% standard NETs). **(C)** Histone H3 citrullinated (absorbance 450 nm). NS, non-significant.

We did not find any significant difference between patients initially hospitalized for moderate COVID-19 disease (n = 46) who later developed ARDS (n = 9) and those who did not (n = 37) [respectively, cell-free total DNA concentrations of 193 ng/ml (177–226) vs. 240 ng/ml (191–290), p = 0.14; MPO–DNA: 0.60 ST (0.40–2.2) vs. 1.2 ST (0.60–2.3), p = 0.58; and H3Cit OD measures: 0.21 (0.081–0.23) vs. 0.16 (0.10–0.31), p = 0.78] (**Figures 2A–C**).

### Levels of Neutrophil Extracellular Trap Markers Are Higher in COVID-19 Patients Who Will Not Survive, Even in the Subgroup of Patients Admitted With ARDS

**Figure 3:**
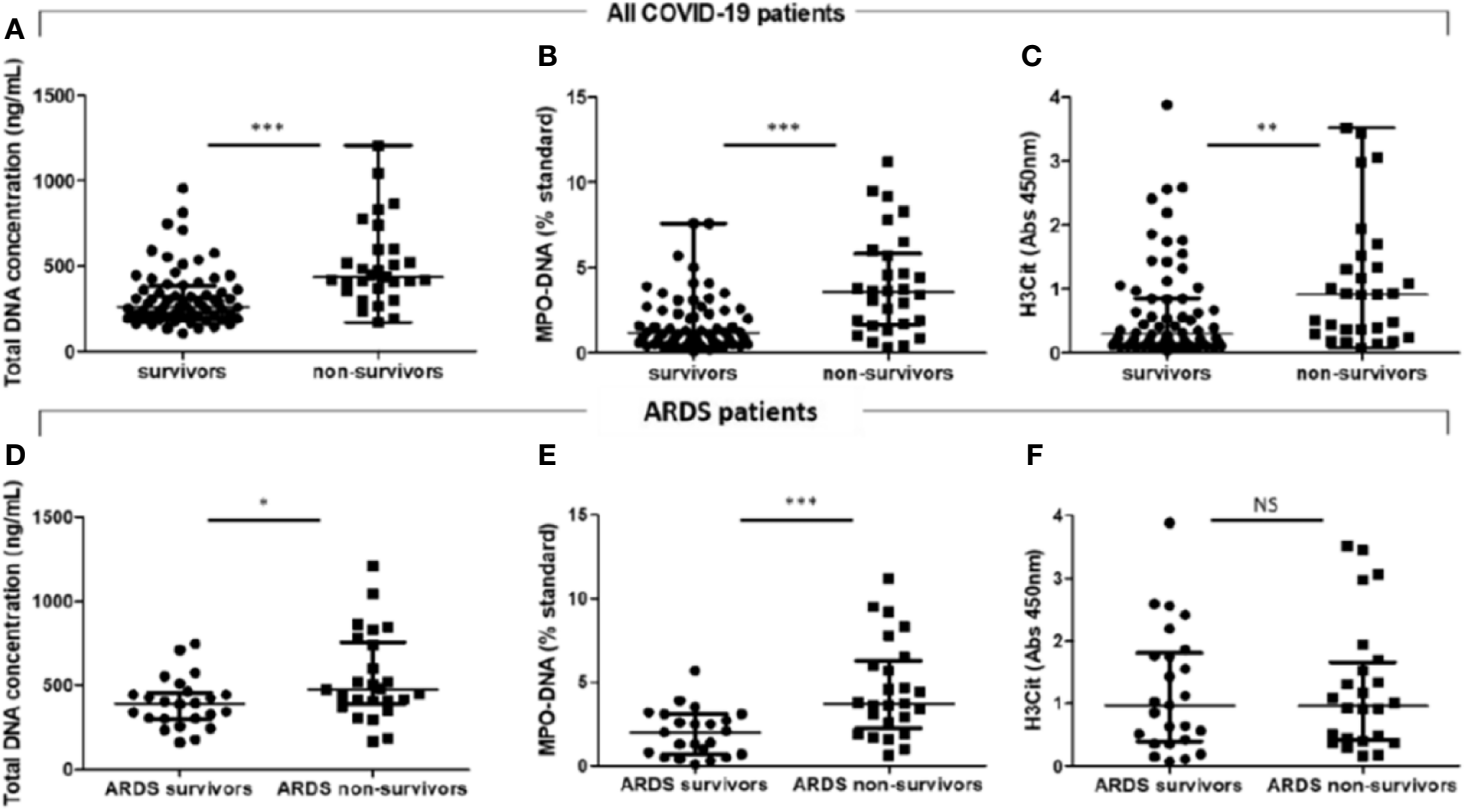
COVID-19 acute respiratory distress syndrome non-survivors have higher plasma total DNA concentrations and myeloperoxidase–DNA levels than survivors. Plasma NET levels were compared in all COVID-19 patients between survivors (n = 67) and non-survivors (n = 29). **(A)** Total DNA concentration (ng/ml). **(B)** Myeloperoxidase–DNA levels (% standard NETs). **(C)** Histone H3 citrullinated (absorbance 450 nm). Plasma NET levels were compared between COVID-19-related acute respiratory distress syndrome (ARDS) survivors (n = 25) and non-survivors (n = 25). **(D)** Total DNA concentration (ng/ml). **(E)** Myeloperoxidase–DNA levels (% standard NETs). **(F)** Histone H3 citrullinated (absorbance 450 nm). Threshold for statistical significance was a p-value of 0.05. *p < 0.05, **p < 0.01, ***p < 0.001. NS, statistically non-significant.

We found that the plasma levels of the 3 NET markers were higher in non-survivor COVID-19 patients than those in survivors [respectively, cell-free total DNA concentration: 437 ng/ml (362–600) vs. 264 ng/ml (200–382), p < 0.0001; MPO–DNA: 3.60 ST (1.65–5.85) vs. 1.20 ST (0.68–2.52), p < 0.001; and H3Cit OD: 0.91 (0.33–1.43) vs. 0.30 (0.14–0.85), p < 0.01] (**Figures 3A–C**). Given that our population of COVID patients includes patients who arrive at the hospital with either moderate or severe disease, we were concerned that analysis of the whole population induces a bias, as the patients who arrive at the hospital with a severe form have *de facto* a worse prognosis than the ones who arrive with a moderate disease. We thus analyzed both populations separately.

In COVID-19 moderate patients, only total DNA concentration, but not MPO-DNA nor H3Cit levels, was higher in non-survivors than that in survivors (**Supplementary Figure S1**), but the low number of events deters from firm conclusions.

We then focused on the more severe patients with the higher mortality rate, i.e., COVID-19-related ARDS patients (n = 50). Cell-free total DNA concentrations and MPO–DNA complex levels measured within the first 3 days after ICU admission were significantly higher in non-survivors (n = 25) than those in survivors (n = 25) (respectively, cell-free total DNA concentration: 475 ng/ml (391–760) vs. 393 ng/ml (303–460), p = 0.03; MPO–DNA: 3.70 ST (2.25–6.25) vs. 2.00 ST (0.70–3.10), p < 0.001] but not plasma H3Cit levels [H3Cit OD: 0.97 (0.41–1.66) vs. 0.97 (0.39–1.81), p = 0.91] (**Table 2** and **Figures 3D–F**). Median time between dosage of NET markers and death was 5 days [3-13]. We did not observe any correlation between plasma levels of NET markers and time to death (**Supplementary Figure S2**).

**Table 2:** COVID-19-related ARDS patients’ characteristics at the time of blood sampling.

	Survivors	Non-survivors	p-value
	N = 25	N = 25	
Age (years)	60 [54–70]	62 [52–69]	0.99
Sex (male)	17 (68%)	17 (68%)	1.00
Body mass index (kg/m²)	28 [25–38]	31 [27–36]	0.65
Hypertension	13 (52%)	18 (72%)	0.48
Diabetes mellitus	5 (20%)	11 (44%)	0.15
Chronic kidney disease	0 (0%)	0 (0%)	1.00
Chronic heart disease	5 (20%)	2 (8%)	0.39
Chronic obstructive pulmonary disease	1 (4%)	11 (44%)	<0.01
Immunosuppressive drug before COVID-19	3 (12%)	8 (32%)	0.39
SOFA	4 [2–9]	5 [2–8]	0.99
PaO_2_/FiO_2_ (mmHg)	149 [115–275]	160 [86–212]	0.39
Fibrinogen (g/L)	7.6 [6.7–9]	7.5 [5.1–8.5]	0.39
D-dimers (mg/L)	1,342 [751–3,760]	3,450 [1,930–8,850]	0.05
Platelets (/mm^3^)	294,000 [178,000–393,000]	166,000 [107,000–297,000]	0.06
Neutrophils (/mm^3^)	5,240 [4,100–9,000]	7,850 [4,430–13,980]	0.26
Lymphocytes (/mm^3^)	840 [680–1,400]	750 [325–1,025]	0.12
Albumin (g/L)	23 [18–26.8]	25 [18–28.5]	0.64
C-reactive protein (mg/L)	137 [77–229]	118 [69–267]	0.88
Invasive ventilation	19 (83%)	25 (100%)	0.27
ECMO	1 (4%)	8 (32%)	0.005
Corticosteroids	8 (32%)	10 (40%)	0.72
Immunomodulating agents	4 (16%)	4 (16%)	1.00
Antiviral agents	8 (32%)	9 (36%)	1.00

Continuous variables are presented as median and interquartile range and are compared using Mann–Whitney test. Categorical variables are expressed as the number of patients (percentage) and are compared using Fisher’s exact test. ARDS, acute respiratory distress syndrome; ECMO, extracorporeal membrane oxygenation; MPO–DNA, myeloperoxidase–DNA; SOFA, sequential organ failure assessment score; %ST, % Standard NETs.

When going back to the clinical and biological characteristics of non-survivor and survivor ARDS patients at inclusion, we did not observe major significant differences except a higher proportion of patients with chronic obstructive pulmonary disease (COPD) and higher levels of plasmatic d-dimers in non-survivors compared to survivors (**Table 2**).

### Levels of Neutrophil Extracellular Trap Markers Are Higher in COVID-19 Patients Who Will Subsequently Develop Pulmonary Embolism Compared With Those Who Will Not, but Not When Assessed in the Subset Group of ARDS Patients

**Figure 4:**
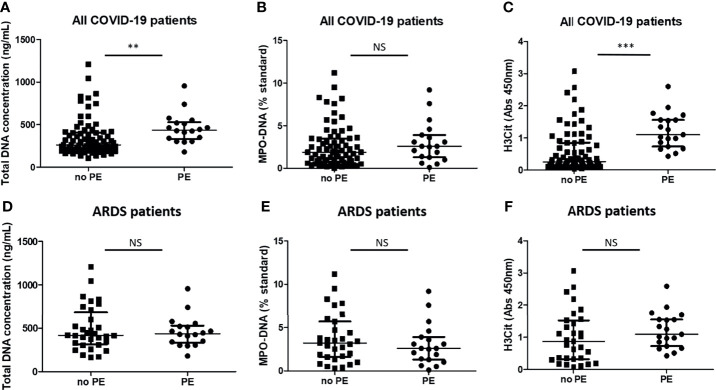
Plasma total DNA concentrations and myeloperoxidase–DNA levels are higher in COVID-19 patients who will subsequently have pulmonary embolism but not when assessed by comparable severity. Plasma NET levels were compared between COVID-19 patients who will subsequently develop pulmonary embolism (PE) (n = 19) and those who will not (n = 77). **(A)** Total DNA concentration (ng/ml). **(B)** Myeloperoxidase–DNA levels (standard NETs). **(C)** Histone H3 citrullinated (absorbance 450 nm). Plasma NET levels were compared between COVID-19-related acute respiratory distress syndrome patients who will subsequently develop pulmonary embolism (PE) (n = 18) and those who will not (n = 32). **(D)** Total DNA concentration (ng/ml). **(E)** Myeloperoxidase–DNA levels (standard NETs). **(F)** Histone H3 citrullinated (absorbance 450 nm). **p < 0.01, ***p < 0.001, NS, statistically non-significant.

Besides being implicated in microthrombus formation leading to lung damage, NETs are also involved in thrombosis in the macrocirculation, especially in veins (21, 22). Consistent with data in conditions other than COVID-19, we found that cell-free total DNA in plasma and H3Cit levels were higher in COVID-19 patients who subsequently developed PE (n = 19) than those who did not (n = 73) [respectively, cell-free DNA concentrations: 437 ng/ml (333–529) vs. 263 ng/ml (198–409), p < 0.01; H3Cit OD: 1.09 (0.72–1.55) vs. 0.26 (0.14–0.85), p < 0.0001] (**Figures 4A, C** **)**. Plasma MPO–DNA levels were not different [2.60 ST (1.30–3.90) vs. 1.90 ST (0.73–3.50), p = 0.27] (**Figure 4B**). PE occurred 4 days (3–6) after the time of sampling [8 days (6–11) after admission].

PE is of particular concern in COVID-19 ARDS patients, as it occurs in about 20% of them (23) vs. only in 3.1% of non-critically ill patients (24). Consistent with these data, only 1 patient in the moderate COVID-19 group subsequently developed PE, 5 days before he died. We thus focused our analysis on those patients (n = 50), and neither plasma cell-free DNA concentrations, MPO–DNA, nor H3Cit levels were different between ARDS patients who later developed PE (n = 18) and those who did not (n = 32) (**Figures 4D–F**). The occurrence of PE did not seem to be associated with death in this subset of critical patients, as 7/25 (28%) non-survivors developed PE vs. 11/25 (44%) in survivors (p = 0.38). No patient died from severe gas exchange impairment or circulatory failure attributed to PE. As D-dimers are a classical biomarker of venous thrombosis, we assessed the correlation between plasma levels of NET markers and D-dimers. Interestingly, only MPO–DNA, but not plasma total DNA concentration nor H3Cit, correlated with D-dimer levels in ARDS patients but with a poor correlation coefficient (r = 0.43) (**Supplementary Figure S3**).

## Discussion

The prediction of disease evolution is a challenge among COVID-19 patients and especially among the most severe ones, i.e., with COVID-19-related ARDS. Our study was designed to assess the involvement of circulating markers of NETs for COVID-19 evolution among inpatients who were admitted at the hospital for moderate and severe COVID-19. We thus included 46 COVID-19 patients with moderate disease and 50 with ARDS. We studied the association between 3 circulating markers of NETs measured shortly after hospital admission and disease evolution in terms of survival, aggravation (for patients with moderate disease only), and PE occurrence. Our findings confirm previous reports showing that circulating markers of NETs correlate with the clinical severity at the time of blood sampling (12–14), but we also report an association between circulating markers of NETs and later survival within the subgroup of patients admitted to the hospital for severe COVID-19 (ARDS patients). The study of Ng et al. (14) previously reported an association between circulating markers of NETs and clinical outcome in a cohort of 106 patients with moderate to severe COVID-19 patients, but this study did not analyze the prognostic value of circulating markers of NETs specifically in each group of patients. Here we did not report any significant association between circulating markers of NETs and disease evolution in the moderate COVID-19 patients in terms of clinical aggravation (i.e., transfer to ICU, PE occurrence, or death). On the contrary, we did find an association between plasma levels of NET markers and survival in ARDS patients.

These results reinforce the hypothesis that immunothrombosis and in particular NETosis are involved in the complications of COVID-19, especially in the most severe forms (13, 25–27). Only experiments in animal models could definitely prove the pathogenic role of NETosis in COVID-19 progression but, at present, several lines of evidence show a link between severe acute respiratory syndrome coronavirus 2 (SARS-CoV-2) infection and NET formation. First of all, several studies reported induction of NET release during COVID-19 either by the virus itself (28), plasma and serum from severe COVID-19 patients (probably through the hyperinflammation typical of severe forms of COVID-19), activated platelets from severe patients (12, 13, 29), and anti-phospholipid antibodies (30). Second, a recent study compared lung specimens from four patients who died from COVID-19 and four from a COVID-19-unrelated cause. The authors reported that NETs infiltrated the lung airways and interstitial and vascular compartments only in severe COVID-19 patients but not in controls, supporting the hypothesis that NETs may drive severe pulmonary complications of COVID-19 (31). The third range of evidence comes from the known role of NETs in thrombosis (32) and the observation that severe SARS-CoV-2 infection induces a prothrombotic state manifesting especially with microthrombosis (33). In line with that, a consortium of authors recently proposed that exaggerated immunothrombosis, occurring for the most part within lung microvessels, drives the clinical manifestations of COVID-19 (32), with the atypical ARDS of COVID-19 being summarized as “microvascular COVID-19 lung vessels obstructive thromboinflammatory syndrome” (MicroCLOTS) (34).

It may look contradictory that we observe an association between circulating NET markers and survival in patients who arrive at the hospital for an already severe form of COVID-19 but that we do not find any association with transfer to ICU in the moderate ones. Part of the explanation could be that host response is still regulated in moderate patients but not in ARDS patients, causing the accumulation of NETs. Another plausible explanation is that NETosis generation could occur mostly within the lung tissue (31, 35) and not the circulation. Lung biopsies are not available in these patients for ethical reasons, as their complications are frequent and possibly lethal.

From a therapeutic point of view, as our results show that NETosis is already highly activated in severe patients who will die, we wonder whether targeting NETosis in severe patients is not already too late. In our opinion, our results suggest that NETosis should be targeted to prevent COVID-19 aggravation, before ARDS occurrence. There are ongoing clinical trials that aim to either prevent NET formation or degrade already-formed NETs. The first ones use anti-inflammatory drugs such as the Janus Kinases 1/2 (JAK1/2) inhibitor ruxolitinib (NCT04338958), dipyridamole (NCT 04391179), and ticagrelor (NCT02735707, NCT04518735). We suspect that these drugs should be more efficient in the less severe patients. DNase I (dornase alpha) can degrade already formed NETs and is currently tested by inhalation in patients with COVID-19 (NCT04402944, NCT04355364, NCT04432987, NCT04359654, NCT04445285, NCT04402970). A major issue with the nebulizing administration route in severe COVID-19 is that there is concern that it will not reach the perialveolar vessels due to the high amounts of platelet factor 4 (PF4) (13) that compacts NETs and decrease their susceptibility to DNase degradation (36).

Thrombosis, and especially PE, is a frequent feature in COVID-19 patients and is an independent risk factor for death (37). It should be noted that the embolic origin of the pulmonary vessel occlusions is questionable, and it may be that the so-called PEs are rather pulmonary thrombi that occur directly in pulmonary arteries (38, 39). Given the known role of NETs in venous thrombosis, we looked for differences in circulating markers of NETs. We found that 2 markers were higher in hospitalized COVID-19 patients who subsequently developed PE than those who did not. But when we restricted our analysis within the specific subgroup of COVID-19-related ARDS patients, who are the ones at more risk to develop PE, we did not find any difference between patients who will develop PE or not. This suggests, if confirmed in a larger cohort, that NETosis in itself is not a major driver for PE (venous thromboembolic event in the macrocirculation). Whereas there is an abundant literature to search for biological markers of clinical aggravation, there are only very few studies that report an association between a biological marker or a clinical parameter that is associated with occurrence of thrombosis among severe COVID-19 patients. D-dimers, which are reported to have a significant predictive value for mortality both in non-critical and critical COVID-19 patients (40, 41), have a limited predictive value for venous thromboembolism (VTE) occurrence, with an area under the curve (AUC) of 0.565 (42).

Our study has several limitations. First, we observe, as others in COVID-19 patients (12), dichotomies between the three circulating NET markers. This could be due to the relatively small number of samples we analyzed and also to the lack of standardization for NET marker measurement. We used 3 different plasma markers to measure NETs. Indeed, despite the discovery of the process of NETosis in 2004 (5), there is still no reference test for NET quantification (6). NETs can be visualized and quantified with conventional fluorescence microscopy, but this assay is hardly reproducible and time-consuming and is better when performed right after blood sampling. To overcome this issue and quantify NETosis that occurs *in vivo*, various plasmatic tests, and mostly ELISAs, have been developed. Given the large amount of tests available and the lack of homogeneity between them, we decided to perform 3 of them: total DNA, MPO–DNA complexes, and H3Cit. Total DNA dosage is not specific for NETs, as it also measures DNA coming from necrotic cells. MPO–DNA complexes are more specific, as it measures DNA together with MPO that specifically comes from neutrophils, but this assay is often not standardized in publications. Here we used a standardized method, with a calibration curve, to allow precise measurement and reproducibility. Lastly, H3Cit measurement can appear to be the most reliable marker, as it directly measures histone H3 that has been citrullinated, a process that is specific from NETosis. But currently, all available ELISAs lack reproducibility and standardization (43). A Scientific and Standardization Subcommittee of the International Society of Thrombosis and Haemostasis is currently running a study aiming at providing recommendation for NETs’ dosage standardization.

Second, computerized tomography pulmonary angiograms were not systematically performed because of in-hospital transport issues regarding these critically ill patients. We only considered here clinically relevant PE. PE occurrence might have been underestimated, as the pretest probability and clinical likelihood of PE could have presumably been lower in patients treated with therapeutic heparin, especially in the ARDS subgroup. Moreover, transportation to CT scan may have been avoided in the more severe patients because of the risks of transferring patients with critical respiratory failure. As catheter-related thrombosis and limb deep-vein thrombosis screening strategy was heterogeneous among centers, we did not analyze those outcomes.

## Conclusion

Taken together, our data demonstrate that circulating markers of NETs are linked to survival but not to PE occurrence in patients with COVID-19 and especially among the most severe ones. Even if measuring NET markers could not be easily implemented in clinical practice to become prognostic biomarkers, our findings are important, as they strengthen the fact that NETosis is a proper therapeutic target in COVID-19 disease, but, more specifically, they argue that NETosis should be targeted before COVID-19 aggravation and ARDS occurrence to be the most efficient.

## Data Availability Statement

The raw data supporting the conclusions of this article will be made available by the authors without undue reservation.

## Ethics Statement

According to French law and the French Data Protection Authority, the handling of these data for research purposes was declared to the Data Protection Officer of the University Hospital of Bordeaux. Patients or relatives were notified about the anonymized use of their healthcare data *via* the departments’ booklets, and non-opposition was recorded. This study was approved by the French institutional authority for personal data protection [Commission Nationale de l’Informatique et des Libertés (CNIL), registration number DEC20-086] and ethics committee (ID-CRB 2020-A00763-36) and by the institutional review board of the University Hospital of Bordeaux (declaration number CE-GP-2020-39). Samples from healthy controls were authorized by the Comité de Protection des Personnes Sud Ouest et Outre Mer III DC 2015/94.

## Author Contributions

RP and CJ designed the study. ADe, ADu, DG, AO, AR, SS, JG, MC, EJ, JP, and GG included the patients and collected the data. SL-C, AD, LG, SS, GG, CJ, and AR collected the samples. SC performed NET measurements. RP, AD, CJ, SC, AD, AR, and SS analyzed the data. RP, AD, and CJ wrote the article. All the authors read and substantially improved the article.

## Funding

This work was funded by a COVID-19 grant from the University of Bordeaux and an ANR-COVID (CORONET R21025GG).

## Conflict of Interest

The authors declare that the research was conducted in the absence of any commercial or financial relationships that could be construed as a potential conflict of interest.

## Publisher’s Note

All claims expressed in this article are solely those of the authors and do not necessarily represent those of their affiliated organizations, or those of the publisher, the editors and the reviewers. Any product that may be evaluated in this article, or claim that may be made by its manufacturer, is not guaranteed or endorsed by the publisher.
