# Supplementary material for: Plasma Markers of Neutrophil Extracellular Trap Are Linked to Survival but Not to Pulmonary Embolism in COVID-19-Related ARDS Patients
Source: Front Immunol. 2022 Mar 17;13:851497. doi: 10.3389/fimmu.2022.851497 (PMC8968169; doi:10.3389/fimmu.2022.851497)
Supplement: Supplementary file 1 [file DataSheet_1.docx]

**Supplemental methods**

**Detailed protocol for MPO-DNA complexes quantification**

A stock solution of NET was made as described previously by Sil *et al* (17)*.* Briefly, we isolated human neutrophils and stimulated them with 100nM of phorbol myristate acetate during 4 hours; NETs were unhooked from cells by mild DNase treatment at 1µg/mL during 15min. We neutralized DNase activity with PBS containing 25mM of EGTA. Cellular suspension was then centrifuged at 4°C, 300g during 5min to pellet the polymorphonuclear cells and collect the supernatant. The supernatant, or stock solution, was aliquoted on ice and stored at−20 °C until later use.

Just before the MPO-DNA complex assay, a working solution of NET was prepared by pooling NET stock solution from 5 independent donors. This solution was considered as 100% NET. A 10-fold dilution in PBS-EGTA 2.5mM was realized, followed by 1:2 serial dilutions in the same buffer to carry out a range of calibration. All the NET-standard solutions were kept on ice until loading in microplate.

We used reagents from the Cell Death Detection Elisa kit (Roche, Basel, Switzerland), and the capture anti-MPO antibody purchased from Bio-Rad. Before starting the assay microplate, all reagents provided in the kit (Coating, Washing and Incubation buffers and ABTS, detection substrate) were brought to room temperature.

The day before the assay, wells were coated with 100µL/well of anti-MPO antibody (5µg/mL in Coating buffer; 4A4 clone, Bio-Rad AbD Serotec, Kidlington, UK) and incubated overnight at 4°C.

The plate was then washed 3 times with washing solution; nonspecific sites were blocked with 200µL of incubation buffer for 30 minutes at room temperature. After washing, 40μL of standard solutions or samples was added to each well, prefilled with 60µL of incubation buffer and incubated for 1.5 h at room temperature. After 3 washes, the wells were incubated for another 1.5 h with 100μL Peroxidase-labeled anti-DNA monoclonal antibody (1:20 in Incubation buffer; clone MCA-33). After 3 washes, 100µL of ABTS (2,2’-azino-di-[3-ethylbenzthiazoline 6-sulfonate]) peroxidase substrate was added in each well and incubated for 30 min at room temperature in the dark and on a shaking device. The reaction was stopped after addition of 100μL of stop solution (Lauryl-sulfate, SDS 1%) and the optical density (O.D.) was measured at a wavelength of 405nm with a reference correction wavelength at 490 nm using a microplate photometer (Infinite® 200 PRO NanoQuant Multimode Microplate Reader from Tecan). We first drew a calibration curve with the optical density values measured for the different dilutions of the 100 % NET standard; the values of MPO-DNA complexes in plasma samples were determined with the NET-standard curve and expressed as percentages of the “NET-standard”.
