# Supplementary material for: Plasma Markers of Neutrophil Extracellular Trap Are Linked to Survival but Not to Pulmonary Embolism in COVID-19-Related ARDS Patients
Source: Front Immunol. 2022 Mar 17;13:851497. doi: 10.3389/fimmu.2022.851497 (PMC8968169; doi:10.3389/fimmu.2022.851497)
Supplement: Supplementary file 2 [file DataSheet_2.docx]

**Supplemental Figure 1 COVID-19 moderate non-survivors have higher plasma total DNA concentrations but not higher plasma MPO-DNA nor H3Cit levels than survivors**

Plasma NET levels were compared in COVID-19 moderate patients between survivors (n: 42) and non-survivors (n: 4).

A: Total DNA concentration (ng/mL). B: Myeloperoxidase-DNA levels (% standard NETs). C: Histone H3 citrullinated (absorbance 450nm).

Threshold for statistical significance was a p-value of 0.05. ** p < 0.001, NS: non-significant.


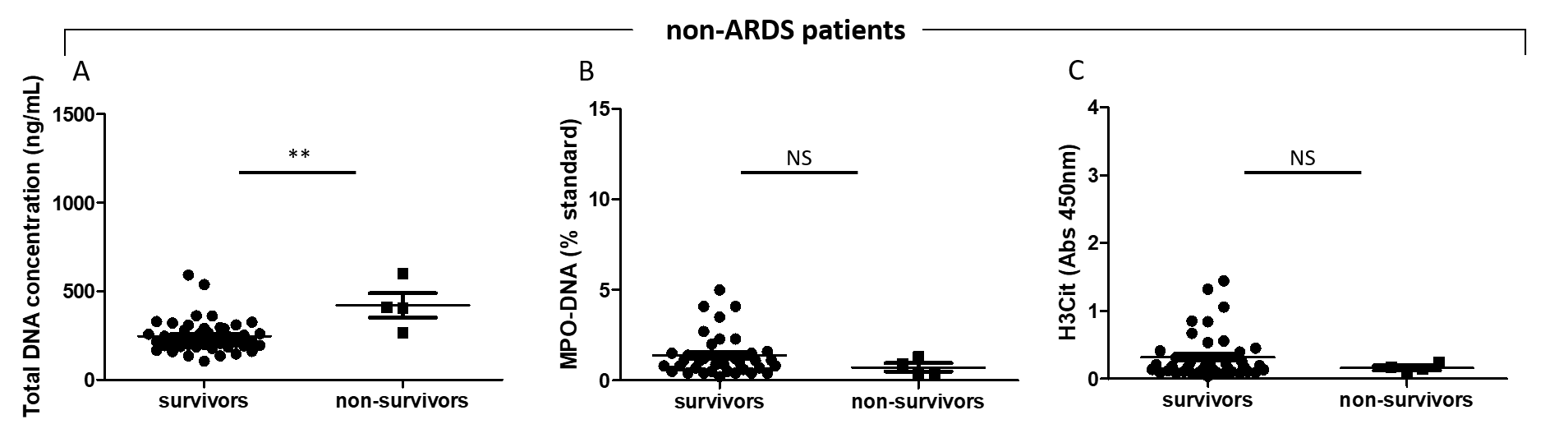


**Supplemental Figure 2 Plasma levels of NET markers did not correlate with time between sampling and death**

Correlation between plasma NET levels and time between sampling and death (days) was assessed by Pearson correlation test in all COVID-19 patients (A, B, C).

A, D: total DNA concentration (ng/mL). B, E: myeloperoxidase-DNA levels (% standard NETs). C, F: Histone H3 citrullinated (absorbance 450nm).

Threshold for statistical significance was a p-value of 0.05.


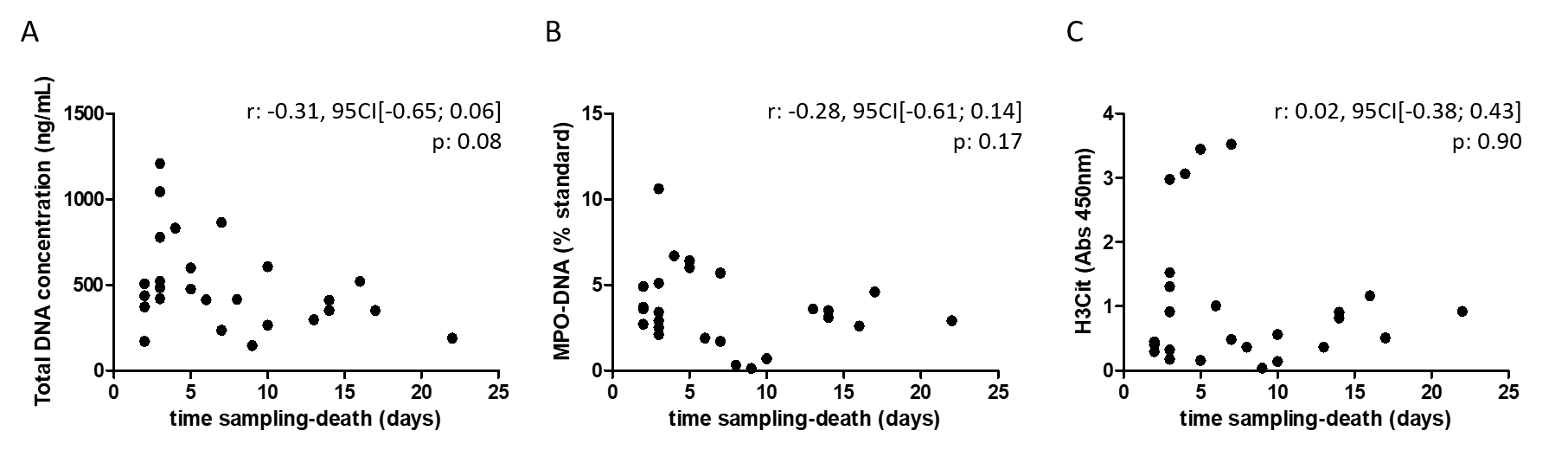


**Supplemental Figure 3 D-dimers poorly correlate with NETs markers**

Correlation between plasma NETs levels and D-dimers was assessed by Pearson correlation test in all COVID-19 patients (A, B, C) and in COVID-19 related ARDS patients (D, E, F)

A, D: total DNA concentration (ng/mL). B, E: myeloperoxidase-DNA levels (% standard NETs). C, F: Histone H3 citrullinated (absorbance 450nm).

ARDS: acute respiratory distress syndrome. Threshold for statistical significance was a p-value of 0.05.

**
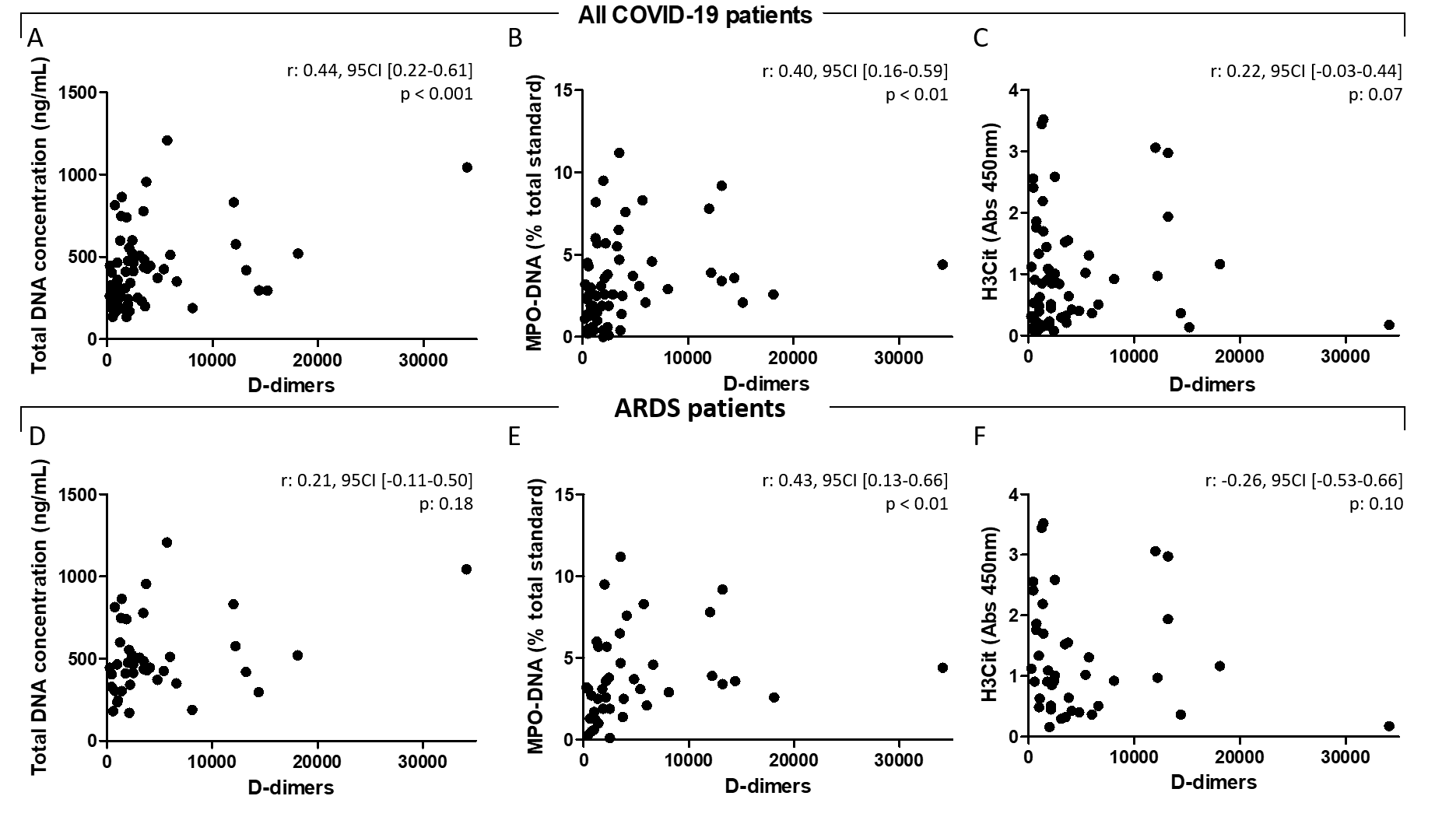
**
